# The non-nuclear DifB NF-κB isoform affects courtship, circadian, and locomotor behavior in adult *Drosophila melanogaster*

**DOI:** 10.3389/fnbeh.2025.1689016

**Published:** 2025-12-10

**Authors:** Thilini P. Wijesekera, Nicole P. Stephens, Aniket Hingnekar, Hanna Y. Gedamu, Natalie R. Dezso, Madison Strange, Saee M. Risbud, Alexander D. Dinh, Nigel S. Atkinson

**Affiliations:** 1 Department of Neuroscience, The University of Texas at Austin, Austin, TX, United States; 2 Freshman Research Initiative, The University of Texas at Austin, Austin, TX, United States; 3 The Waggoner Center for Alcohol and Addiction Research, The University of Texas at Austin, Austin, TX, United States

**Keywords:** NF-κB, NF kappaB, courtship, circadian rhythmicity, learning, synaptic localization, *Drosophila melanogaster*, alternative splicing

## Abstract

The Drosophila *Dif* gene uses alternative messenger RNA (mRNA) processing to encode two different nuclear factor kappa Bs (NF-κBs). The DifA isoform is a canonical NF-κB transcription factor that is important for activation of the immune response. Our primary interest is the DifB isoform, which is neuron-specific and expressed in the mushroom bodies and antennal lobes of the adult brain. The DifB protein lacks a nuclear localization signal and does not enter the nucleus. Instead, it localizes to the cell body surrounding the nucleus, to axonal-dendritic projections, and to the synapse. DifB is an unusual member of the NF-κB superfamily, as it acts outside the nucleus to modulate behavior. The DifB isoform has been shown to modulate the sensitivity of the adult to sedation by alcohol. Here, we conducted a survey to determine whether the DifB NF-κB is important for other fly behaviors. We observed that a DifB-specific mutation strongly suppresses male courtship. However, despite the expression of DifB in the mushroom bodies, a DifB null allele does not interfere with learning in a learned-suppression-of-phototaxis assay. Finally, both DifA-specific and DifB-specific mutations caused flies to have a circadian long rhythm phenotype, although the circadian phenotype cannot be scored in male DifB mutants because of a sexually dimorphic locomotor defect.

## Introduction

In mammals, Toll-like receptor (TLR) pathways are key activators of the innate immune system. A number of these TLR pathways also modulate behaviors relevant to the understanding of alcohol-use disorders. *Drosophila melanogaster* has nine Toll-like receptors (Hu et al., 2011). Among Drosophila Toll-like receptors, the most is known about Toll — the founding member of this receptor class.

The Toll signaling pathway was first discovered based on its embryonic role in dorsal-ventral pattern formation. In embryonic development, the output of the Toll pathway is the nuclear factor kappa B (NF-κB) encoded by the *dorsal* gene (Belvin and Anderson, 1996). In adult flies, the Toll signaling pathway has been repurposed and has been shown to be critical in the response of the innate immune system to infection by gram-positive bacteria and fungi, in setting the behavioral sensitivity to sedation with alcohol (ethanol), and in the performance of neurotrophin receptor-like activity in the brain (Hedengren-Olcott et al., 2004; Troutwine et al., 2016; Zhu et al., 2008).

In adults, the known output of the Toll pathway is not NF-κB encoded by the *dorsal* gene, but instead NF-κB encoded by the *Dif* gene. The linkage between Toll activation and Dif signaling has been demonstrated in the adult immune system and in the adult nervous system (Troutwine et al., 2016; Hoffmann and Reichhart, 2002).

Wijesekera et al. (2022) tested the contribution of the *Dif* gene to immune function and alcohol response. The *Dif* gene encodes two protein variants generated by alternative mRNA splicing (Zhou et al., 2015). These are referred to as DifA and DifB. The DifA protein isoform was shown to be needed for normal immune function, while the DifB protein isoform was shown to be required for normal sensitivity to sedation with alcohol. With regard to the alcohol sensitivity phenotype, DifB was connected to the Toll pathway by demonstrating that up- or down-expression of any step in the signaling pathway reduced or increased ethanol sensitivity, respectively. In addition, an epistatic relationship between the gene that encodes the top of the pathway (*Toll*) and the bottom of the pathway (*Dif*) was shown to exist (Troutwine et al., 2016; Wijesekera et al., 2022). Neither mutation in DifA nor DifB influenced the preference for consumption of food laced with 5% ethanol.

In the adult brain, the DifB isoform was shown to be expressed in neurons of the mushroom bodies, antennal lobe, and, very weakly, in the subesophageal ganglion. The DifA isoform is not expressed in the nervous system but is expressed in the immune system. Interestingly, the neural-specific DifB isoform lacks a nuclear localization signal and does not enter neuronal nuclei. Instead, it has been shown to localize to the synapse. The mushroom bodies are known to be important in the response of Drosophila to alcohol (Ghezzi et al., 2013; Larnerd et al., 2025; Xu et al., 2012) and to play an important role in Drosophila learning and memory (Davis, 1993). Therefore, it is possible that the absence of DifB expression in mushroom bodies in the DifB hypomorph is the cause of changed alcohol sensitivity in DifB hypomorphs and that DifB mutations might also affect learning and memory in flies.

In mammals, NF-κBs are also found at neuronal synapses in the hippocampus, where they have been shown to be important in learning and memory (Dresselhaus and Meffert, 2019; Salles et al., 2015). These mammalian synaptic NF-κBs are believed to be stimulated by neural activity to translocate to the nucleus where they alter gene expression (Mikenberg et al., 2007). However, the Drosophila DifB NF-κB is unique in that it is the only NF-κB reported to not be able to enter the nucleus and thus act only outside of the nucleus. We hypothesize that DifB influences behavior by directly modulating synaptic activity. Because of its novel localization at the synapse in the mushroom bodies, we sought to determine whether DifB participates in other behaviors beyond its role in sensitivity to sedation with alcohol.

## Results

**Figure 1:**
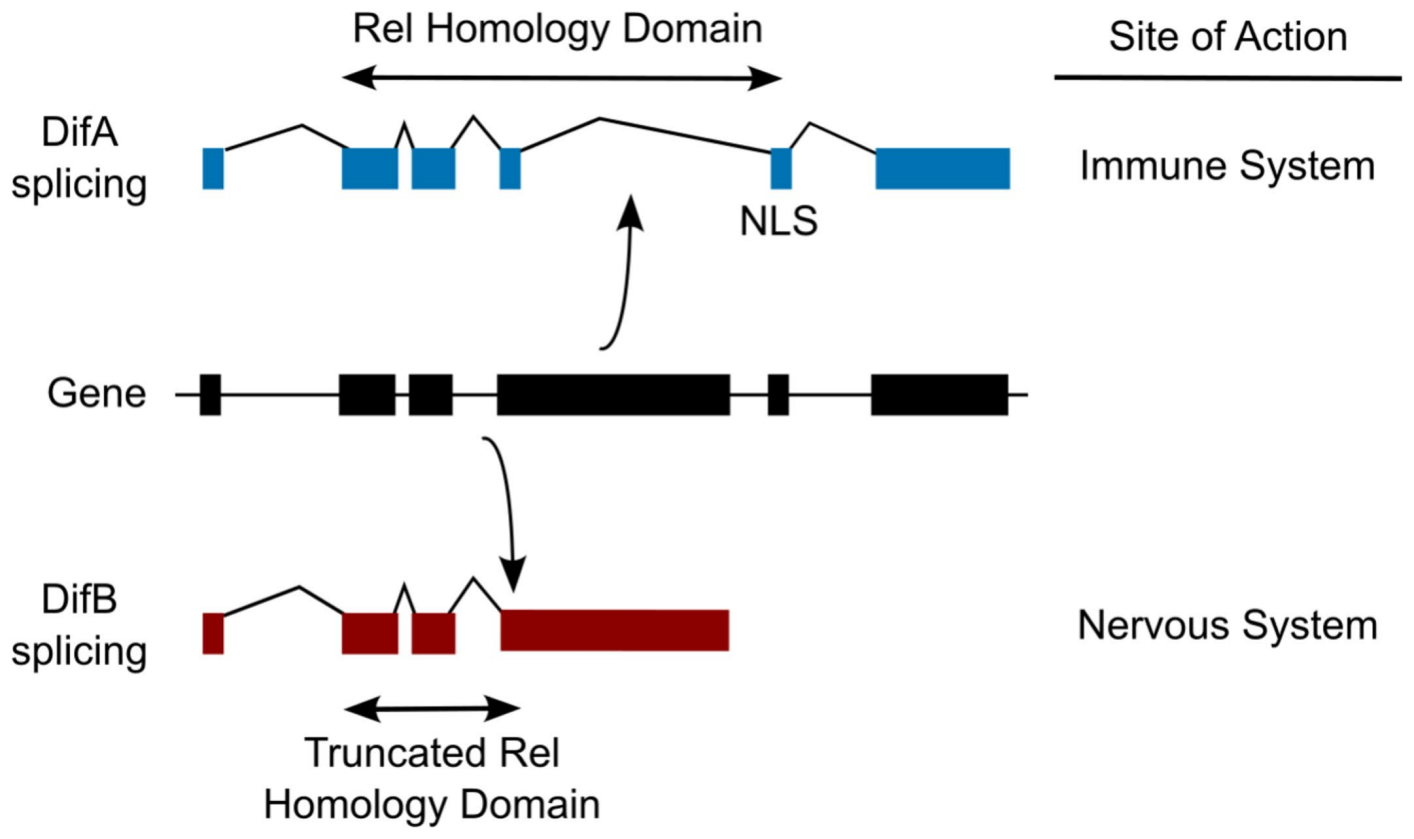
Alternative mRNA splicing generates transcripts that encode two distinct protein isoforms, DifA and DifB. DifA is a canonical NF-κB with a full Rel Homology domain (RHD) and a nuclear-localization signal (NLS). DifB is missing part of the RHD and the NLS. DifA is 667 amino acids (74 kDa; UniProt Accession Number: E1JHK1; Flybase names are Dif-PA, PB, and PD), while DifB is 987 amino acids in length (108 kDa; UniProt Accession Number: E1JHK2; Flybase name is Dif-PC). The Rel Homology Domain is as identified by Uniprot.org. Details of the construction of the mutant alleles and their validation are provided in the Methods section.

Alternative messenger RNA (mRNA) processing of *Dif* gene transcripts generates two protein isoforms called DifA (expressed in adult immune cells) and DifB (expressed in the adult central nervous system) (Figure 1). Here, we ask whether the *Dif* gene modulates other commonly studied behaviors.

### DifB but not DifA is required for normal courtship behavior in male flies

**Figure 2:**
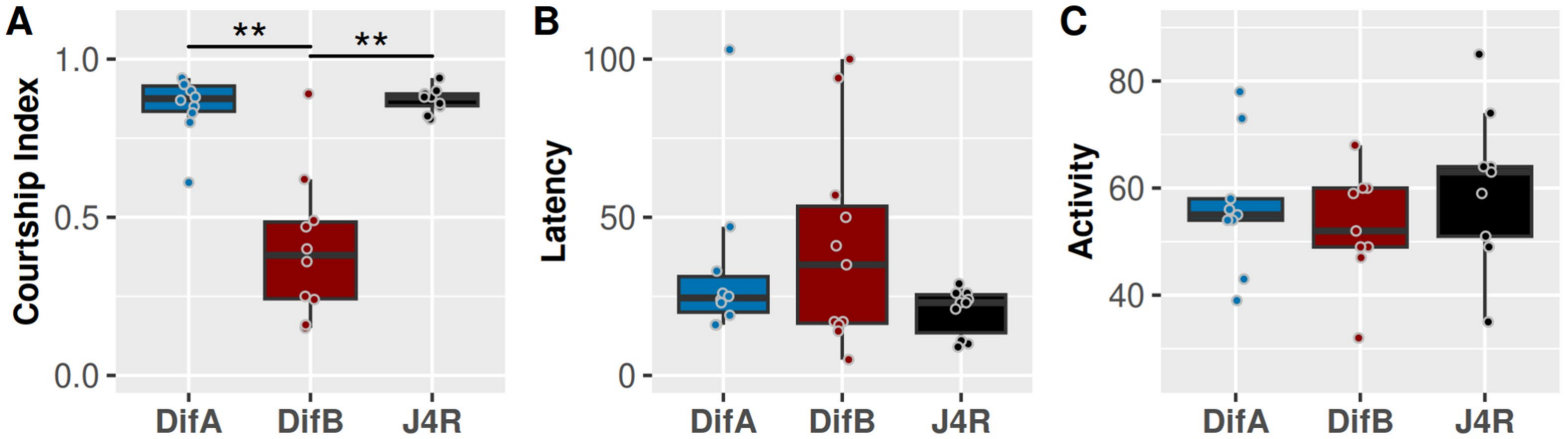
DifB protein is required for normal courtship behavior in flies. **(A)** DifB mutant flies have a reduced courtship index compared to DifA mutant and the genetically matched control (J4R) flies. (Kruskal-Wallis 𝛸_2_ = 14.354, df = 2, *p* = 0.000764, *N* = 10; Bonferroni-corrected Mann–Whitney *post hoc* tests indicate DifA vs. J4R *p* = 1; DifB vs. J4R *p* = 0.003862467; DifA vs. DifB *p* = 0.003928269). **(B)** Dif mutants and the J4R control line have comparable latency to initiation of courtship (seconds to the initial orientation of the male to the female; Kruskal-Wallis 𝛸_2_ = 1.9005, df = 2, *p* = 0.3866, *N* = 10 or 11; Bonferroni-corrected Mann–Whitney *post hoc* tests indicate DifA vs. J4R *p* = 0.7651355; DifB vs. J4R *p* = 0.734454; DifA vs. DifB *p* = 1). **(C)** DifA and DifB do not appear to differ from the J4R control line with respect to their level of locomotor activity (Kruskal–Wallis 𝛸_2_ = 1.6769, df = 2, *p* = 0.4324, *N* = 9; Bonferroni-corrected Mann–Whitney post hoc tests indicate DifA vs. J4R *p* = 1; DifB vs. J4R *p* = 0.5963442; DifA vs. DifB *p* = 1). Most samples were not normally distributed (Shapiro–Wilk normality test), and so we used the Kruskal–Wallis rank–sum test and the Bonferroni-corrected Wilcoxon rank sum test with continuity correction post hoc tests to determine significance.

We performed 10-min male courtship assays comparing the DifA mutant and DifB mutant and the genetically matched control line called J4R. The DifA and DifB mutations eliminate their respective transcripts and proteins but do not alter the expression of the other Dif isoform (Wijesekera et al., 2022; Zhou et al., 2015). The courtship index accounts for the total time during which any element of courtship behavior was displayed (Siegel and Hall, 1979). The DifA mutation showed a similar courtship index and latency to court as the J4R control. However, the DifB mutation strongly suppressed male courtship (Figure 2A). No difference in latency to court was observed in the DifB mutant (Figure 2B). No difference in the gross locomotor activity, as measured in the circular courtship assay chamber, was observed (Figure 2C).

### Neither mutations in DifA or DifB alter female receptivity or the incidence of male: male courtship

**Figure 3:**
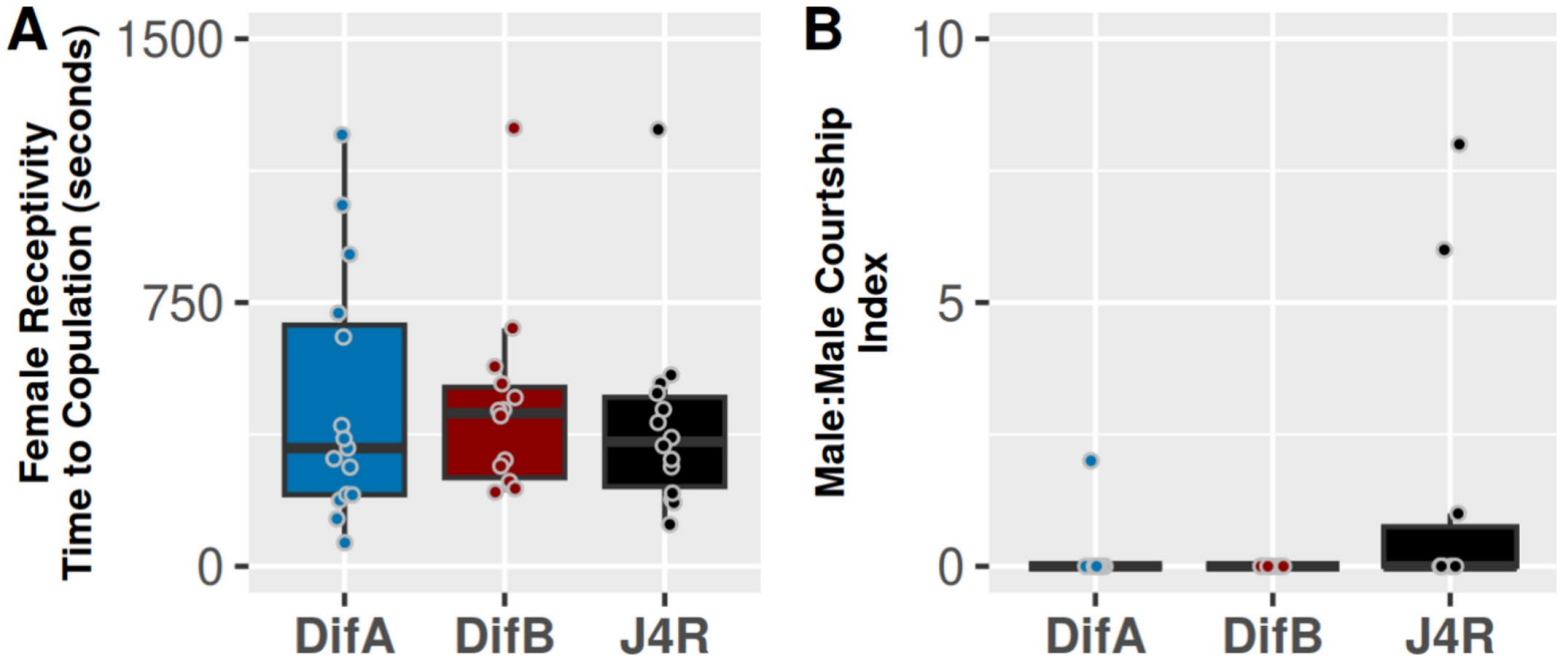
**(A)** Neither the DifA mutation nor the DifB mutation significantly alters female receptivity. Y-axis is seconds to copulation (Kruskal-Wallis 𝛸_2_ = 0.45739, df = 2, *p*-value = 0.7956, *N* = 14–16; Bonferroni-corrected Mann–Whitney *post hoc* tests indicate DifA vs. J4R *p* = 1 and DifB vs. J4R *p* = 1). **(B)** Neither DifA nor DifB mutations significantly enhance male–male courtship as measured by the wing-extension index of the males. (Kruskal–Wallis 𝛸_2_ = 5.8173, df = 2, *p*-value = 0.05455, *N* = 10–11; Bonferroni-corrected Mann–Whitney *post hoc* tests indicate DifA vs. J4R *p* = 0.2569394 and DifB vs. J4R *p* = 0.0904262). Most samples were not normally distributed (Shapiro test), and so we used the Kruskal–Wallis rank-sum test and Bonferroni-corrected Mann–Whitney *post hoc* tests to determine significance.

We explored the potential of the Dif protein isoforms to influence female receptivity. Female receptivity assays were performed using 5-day-old DifA mutant females, DifB mutant females, and J4R control females. All males were from a Canton S wild-type line. Each female fly was placed in a courtship chamber for 2 min, after which an isolated virgin male was introduced. The assay was carried out for 60 min, and the time period to copulation was recorded. We did not observe a difference in latency to copulation for any of the females, indicating that Dif proteins do not influence female receptivity (Figure 3A). Male:male courtship has been shown to be triggered by mutations in a variety of genes (e.g., expressing TRPA, Myc, or White) or treatments (e.g., increasing dopamine levels, intense light, and even daily intoxicating doses of ethanol) (Wang et al., 2011; Liu et al., 2008; Ueda et al., 2023; Zhang and Odenwald, 1995; Pan et al., 2022; Lee et al., 2008). Therefore, we sought to determine whether mutations affecting DifA or DifB enhanced male:male courtship. However, mutations affecting the Dif isoforms also did not alter the incidence of male:male courtship (Figure 3B).

### Circadian rhythms

**Figure 4:**
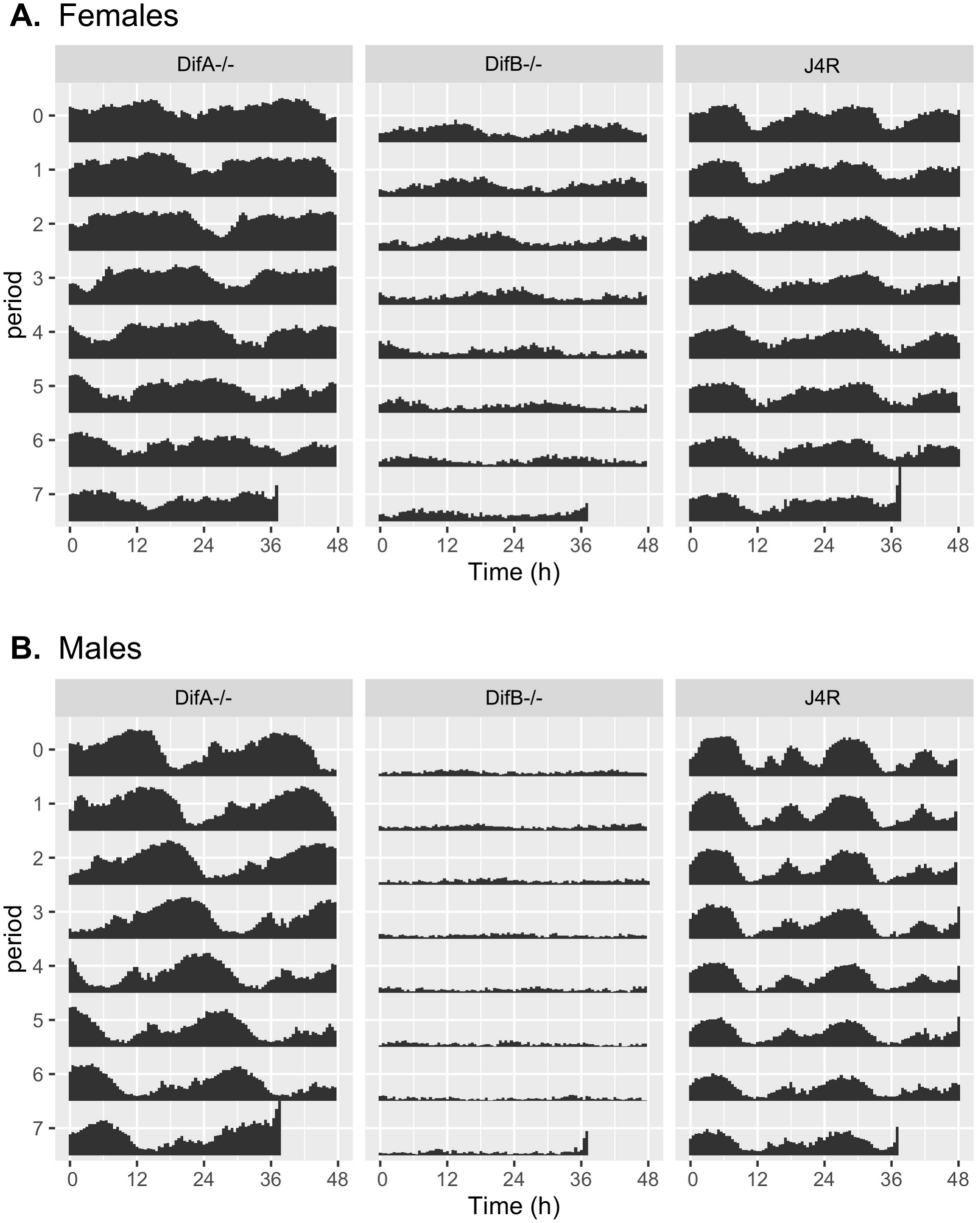
Double-plotted actogram shows differences in rhythms between the *DifA* and *DifB* mutants and the J4R control. **(A)** Female animals shown. *DifA*- homozygous females show a non-24 h rhythm indicated by the progression of activity maxima across the day. *DifB*—homozygotes have drastically reduced activity but still display rhythmicity. The J4R *Dif* wild-type control shows activity cycles that appear to have a 24-h periodicity. These are the same data as in Figure 5, where circadian period, number of repeats, and statistical analysis of the data are given. **(B)** Male animals are shown. Actogram of *DifA* mutant males shows strong rhythms with a clear, greater than 24 h period. *DifB* mutants exhibit very low locomotor activity. A clear periodicity to the remaining rhythm is not obvious. J4R males wild type for *Dif* show a rhythm with clear 24-h periodicity. These are the same data as in Figure 5. The number of repeats and statistical information are described in the legend for Figure 5.

**Figure 5:**
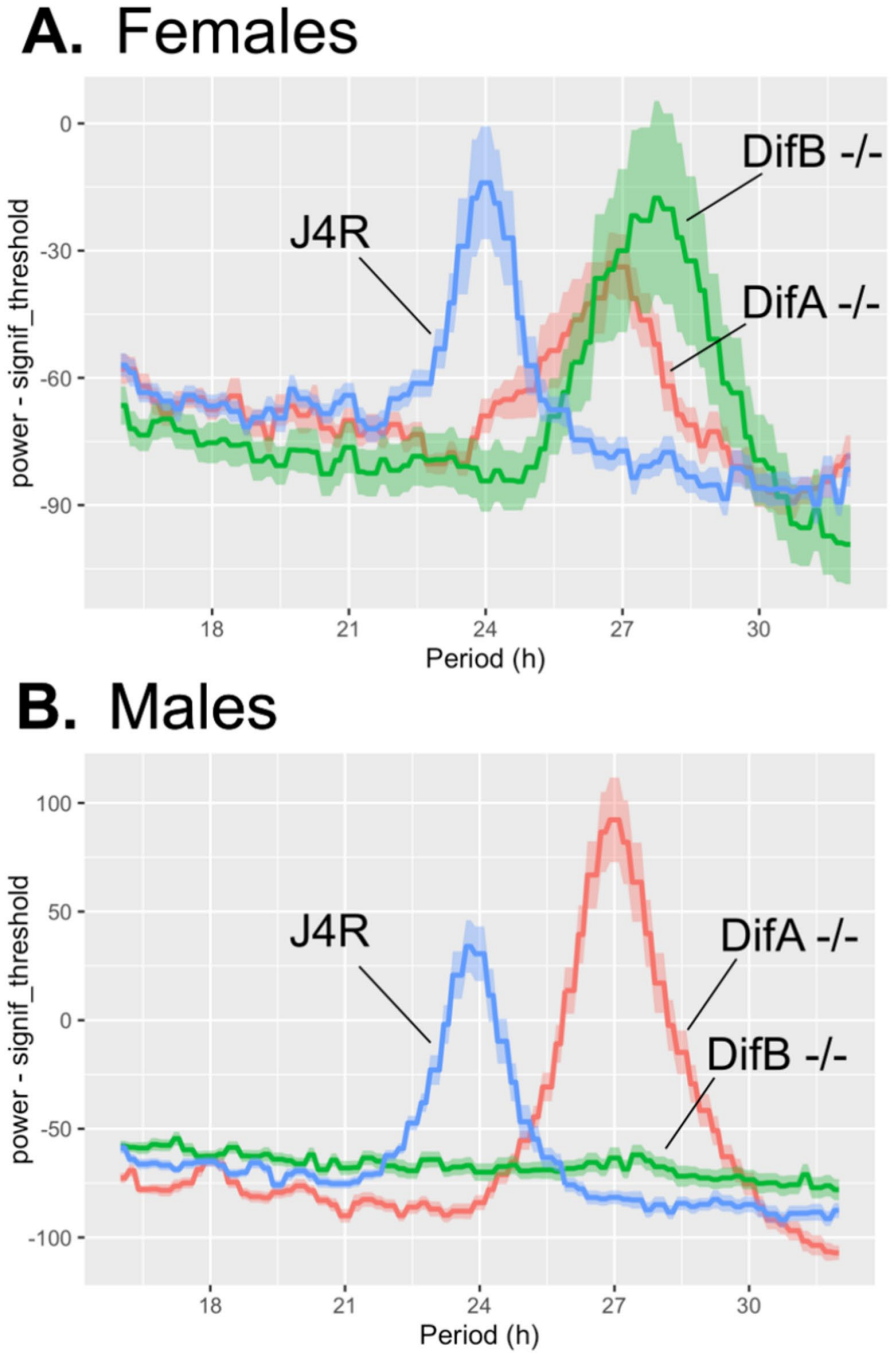
The population average periodogram shows that the *Dif* mutations affect circadian rhythms. The line represents the mean of the movement at each 1 min interval. The vertical error bars are SEM. **(A)** Female *DifA* and *DifB* mutants exhibit a long phenotype, with a mean rhythm of 26.42 ± 0.29 h (SEM) and 27.74 +/− 0.15 h, respectively. The J4R control displayed a rhythm of 23.89 +/− 0.14 h. Both female *DifA-* and *DifB-* homozygotes differ significantly from the J4R control (*p* = 0.0192 and *p* = 0.0028, respectively). *DifA-* and *DifB-* also differ from each other with a *p* = 0.0192. **(B)** The male *DifA* mutant and J4R control animals have rhythms of 26.89 ± 0.06 h and 23.62 +/− 0.07 h, respectively. The Rethomics software did not report a rhythm for *DifB* mutant males. The *p*-values are pairwise Wilcoxon rank–sum tests. *N* = 21–22 for females and 32 for males. Only one male and eight females died during the assay period and were therefore excluded from the analysis. Animal death was not obviously associated with any given phenotype. All assays were conducted over the same 7-day period in darkness in the same incubator.

As shown in Figures 4 and 5, and Table 1, the mutation that eliminated DifA expression caused both females and males to have a long rhythm with a period of 26.42–26.89 h. DifB mutant females also exhibit a long rhythm phenotype showing a circadian rhythm of 27.74 h. DifB mutant females also show some reduction in locomotor activity. DifB mutant males, in contrast, showed such a large reduction in locomotor activity that the analysis software failed to detect circadian periodicity in their activity. The lack of activity is not due to a loss of viability, as only 1 of 32 males tested died during the assay.

**Table 1:** Mean circadian rhythm periodicity in hours with SEM.

Genotype	DifA −/−	DifB −/−	J4R
Females	26.42 +/− 0.29	27.74+/− 0.15	23.89 +/−0.14
Males	26.89 +/− 0.06	None detected	23.62 +/− 0.07

### Learning and memory

**Figure 6:**
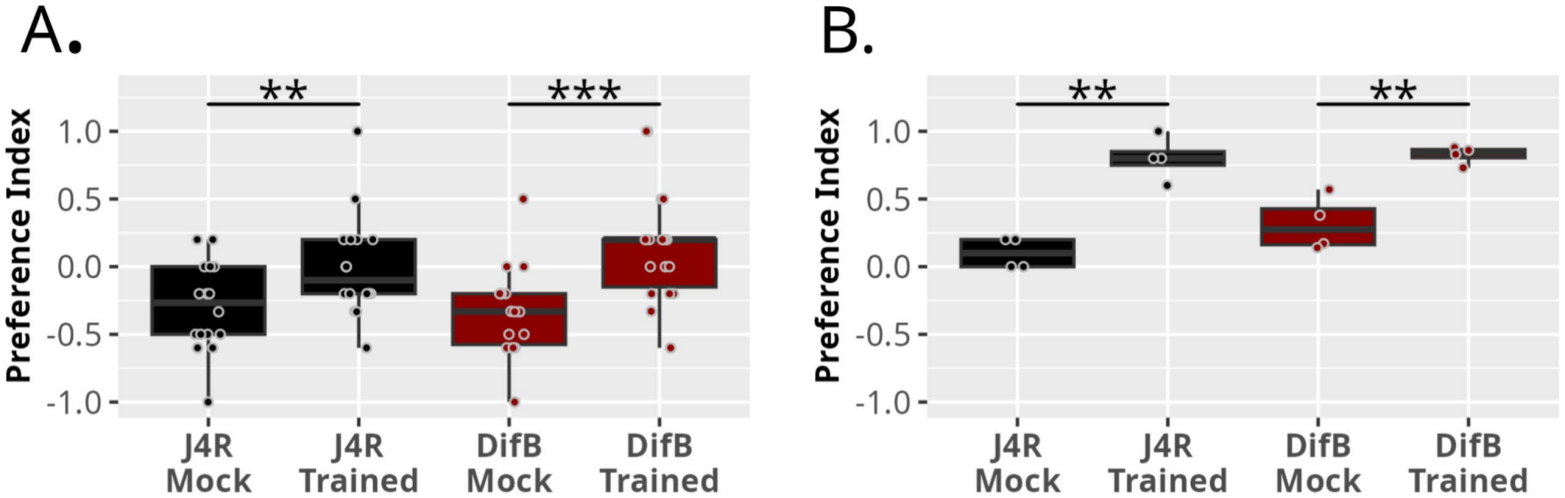
DifB does not affect the capacity to learn and remember in the learned-suppression-of-phototaxis assay. **(A)** Males kept in isolation prior to eclosion were entrained to avoid white light illumination that had been repeatedly paired with quinine exposure. Flies are placed in a choice chamber in which they can choose a light or dark arm. A more positive preference index indicates that the flies more often choose the darkened arm. Each data point (*N*) represents a preference index determined for 3–5 flies. Data were normally distributed (Shapiro test) and had equal variance (F test) so we used a two-tailed Student’s *t*-test to calculate *p* values. J4R control showed a modest capacity for learning in this assay (*p* = 0.02481685; *N* = 16). DifB mutant also showed a modest capacity to learn (*p* = 0.0005242034; *N* = 18). **(B)** DifB also does not obviously compromise female learning in this assay. Data had equal variance (F test) but not all data points were normally distributed. Therefore we used the Mann–Whitney Wilcoxon Test. The J4R stock learned well (*p* = 0.02746864; *N* = 4) and the DifB stock learned well (*p* = 0.02857143; *N* = 4).

DifB is strongly expressed in the adult mushroom bodies, an insect brain structure of central importance in learning and memory, whereas DifA is not expressed in the adult brain (Wijesekera et al., 2022). Therefore, we asked whether the loss of DifB expression affected learning (Figure 6). The capacity for associative learning was tested using a learned-suppression-of-phototaxis assay (Le Bourg and Buecher, 2002; Seugnet et al., 2009; Troutwine et al., 2019). Flies are naturally positively phototaxic. In this assay, flies are trained to avoid light by pairing it with exposure to 0.1 M quinine-soaked paper. Quinine is strongly aversive to flies. The preference for dark is then measured using a darkened T-maze in which one path leads to a lit vial and the other to a dark vial. Untrained or mock-trained animals usually choose the lit vial. After training, most animals show a preference for the darkened vial. Seugnet et al. (2009) demonstrated that learning in this paradigm depends on synaptic activity in the mushroom bodies, that learning is suppressed by mutations in *dnc* or *rut* genes, and that the learning deficit in these mutants can be complemented by mushroom body expression of the respective genes. Thus, this assay is comparable to more commonly used learning assays. The DifB mutation had no effect on the capacity of the mutant to learn in the learned-suppression-of-phototaxis assay.

One should not assume that the differences in the learning profiles of males and females are meaningful. We have observed daily and seasonal differences in preference indices. The male and female assays were performed on different days.

## Discussion

Here, we extend our description of the behavioral contributions of a non-nuclear NF-κB found at neuronal synapses. Localization of NF-κB at synapses has been observed in mammals for a long time (Kaltschmidt et al., 1993). In mammals, these NF-κBs modulate the shape of dendritic spines and synaptic plasticity (Dresselhaus and Meffert, 2019). It has been shown or assumed that they do so by translocation of the NF-κBs into the nucleus, where they can modulate transcriptional activity in ways that modify the spine or synapse. However, it had been proposed that in mammals they might also act locally at the synapse, although this has never been tested, probably because it is difficult to test for local synaptic effects in a background of strong genomic effects (Dresselhaus et al., 2018). For this reason, the Drosophila DifB NF-κB is particularly interesting because it does not have the capacity to enter the nucleus but has retained the capacity to localize to the synapse (Wijesekera et al., 2022). The DifB isoform permits the study of non-nuclear effects of an NF-κB on neural function in the absence of a nuclear contribution. Here, we continue the characterization of a mutation that specifically eliminates expression of the non-nuclear DifB without altering expression of the nuclear DifA isoform (Wijesekera et al., 2022; Zhou et al., 2015).

Previously, showed that mutant DifB homozygotes were more sensitive to sedation by ethanol vapor but showed normal interest in ethanol-laden food (Wijesekera et al., 2022). Here, we demonstrate that the DifB loss-of-function mutation affects other behaviors. The DifB mutant causes males to court very poorly. In addition, we tested the circadian rhythmicity of both mutants. In females, both DifA and DifB mutations produced animals with a long circadian rhythm phenotype. In addition, in males, DifA mutant homozygotes also clearly exhibited a long rhythm phenotype. Unfortunately, the effect of the DifB mutant on male rhythmicity could not be conclusively determined because the animals did not move sufficiently in the circadian rhythm assay. It is not understood why both DifA and DifB mutations affect circadian rhythmicity since our previous immunohistochemical staining for DifA and DifB in the adult and the monitoring of GFP-tagged DifA and DifB isoforms in the adult indicated that only the DifB variant is expressed in the adult brain (Wijesekera et al., 2022). However, in the larval brain, we did previously see low, level ubiquitous expression of DifA in tandem with specific strong expression of DifB in the larval mushroom bodies (Wijesekera et al., 2022). Thus, at an earlier developmental stage, DifA and DifB may be co-expressed, and this could have developmental consequences on the adult that affect adult pacemaker cells.

We were surprised that the DifB mutant males moved so little in the circadian assay, whereas in the courtship assay we saw that DifB mutants had locomotor activity indistinguishable from that of DifA mutants or the control. The courtship assay is performed in a circular chamber while the circadian assay occurs in a narrow tube (5-mm interior diameter). Male locomotor activity was clearly different in circular chambers than in narrow tubes. We speculate that differences in locomotor activity in different-shaped test chambers could indicate that DifB is involved in thigmotaxis; however, a more rigorous study is required to conclude that DifB plays a role in this phenomenon (Soibam et al., 2012). The mushroom bodies are also important for this behavior (Wijesekera et al., 2022; Besson and Martin, 2005).

Although the DifB mutant does not manifest a walking phenotype in the courtship assay, it might be that the lack of courtship activity is actually an incoordination phenotype that, in the absence of close walls, disrupts normal male courtship.

Finally, because DifB is strongly expressed in the fly mushroom body, we also asked whether the DifB mutation affected learning (Wijesekera et al., 2022). Despite DifB showing its strongest expression in the mushroom bodies, no change in learning capacity was observed in this mutant.

How might DifB modulate behavior? Three of the behaviors surveyed in this paper have been linked to normal mushroom body function: male courtship (Lim et al., 2018), consolidation and retrieval of memories (Davis, 1993; Cervantes-Sandoval et al., 2013), and locomotor activity (Martin et al., 1998; Helfrich-Förster et al., 2002). While all are clearly linked to the mushroom bodies, these phenotypes are functionally or genetically separable. For instance, Lim et al. (2018) demonstrated that the D1 dopamine receptor is required in the MB for normal courtship drive in naive males but is not required for robust performance in a learning and memory assay. This group also showed that courtship could be suppressed without obviously affecting locomotor activity. In addition, even though the mushroom body has been shown to act as an inhibitor of locomotor activity and inhibition or ablation of the mushroom bodies results in a substantial increase in locomotor activity (Martin et al., 1998; Helfrich-Förster et al., 2002), mutations in *rut* and *dnc*, well-known mushroom body-acting learning and memory genes, can produce learning deficits without affecting locomotor activity (Zhang et al., 2015). Finally, one can also genetically interfere with mushroom body-dependent learning without depressing the male courtship index (Main et al., 2021).

Previously, we had shown that DifB modulates the sensitivity to alcohol sedation via its action within the Toll pathway. We used an epistasis experiment to show that, with respect to the sensitivity to alcohol sedation phenotype, in neurons the DifB NF-κB is functionally downstream of the Toll receptor (Troutwine et al., 2016). The action of the Toll pathway in neurons that modulate behavior might seem unusual. However, flies reuse Toll and most Toll-like receptors to perform neurotrophin receptor function. In fact, flies do not have canonical neurotrophin receptors (such as orthologs to mammalian Trk, p75NTRP, or Sortilin) (Zhu et al., 2008; Li and Hidalgo, 2021; Atkinson, 2023). Therefore, it is possible that the effect of the DifB mutation on adult fly behavior arises because of a disturbance in Toll-mediated neurotrophin-like signaling. Whether or not mammals also use Toll-like receptors for their neurotrophin receptor-like activity is not yet known (Sun et al., 2024).

This work indicates that, despite the continuous study of NF-κBs since their discovery in 1986 (Sen and Baltimore, 1986), NF-κBs may play unexpected roles, and these may be important for normal behavior. It is also possible that NF-κBs modulate behavior in mammals via non-genomic effects, as observed in flies, but that these effects remain undocumented because of the axiomatic assumption that all NF-κB effects are genomic in nature.

## Methods

### Fly husbandry and fly stocks

Flies were raised on cornmeal malt extract food (7.6% CH Guenther & Son Pioneer Corn Meal) (Walmart, Inc., Bentonville AR, USA), 7.6% Karo syrup (Walmart, Inc., Bentonville AR, USA), 1.8% Brewer’s yeast (SAF, Milwaukee, WI, USA), 0.9% Gelidium agar (Mooragar, Inc., Rocklin, CA, USA), 0.1% nipagin (Fisher Scientific, Inc.) in 0.5% ethanol, 11.1% amber malt extract (#24-1234G, Austin Homebrew, Austin, TX, USA) and 0.5% propionic acid (Fisher Scientific, Inc.). Solids were weight/volume and liquids were volume/volume. Flies were housed in a 12:12 light:dark cycle at ~21 °C. The DifA and DifB mutant flies and the J4R matched control line were obtained from SA Wasserman (University of California San Diego) CA, USA.

The DifA and, DifB mutants, and J4R were constructed by Zhou et al. (2015). To recapitulate, all three are built in a Df(2 L)J4 background which has a 39 kb deletion that removes all of *Dif*. Recombineering-mediated gap repair was used to generate a 43-kb clone spanning the deletion in Df(2 L)J4. This BAC subclone was inserted in an attP landing site at 86Fb to create J4R. The DifA and DifB mutants were built in the exact same manner except that site-specific mutagenesis was first used to eliminate the potential of expressing DifA or DifB in the BAC subclone, respectively (Zhou et al., 2015). These mutant alleles were validated by genomic PCR and genomic DNA sequencing. In addition, the alleles were shown to be protein null alleles. Finally, the DifA mutation did not alter the expression of the DifB mutation and vice versa (Wijesekera et al., 2022). Canton S wild-type animals were also used in courtship assays.

### Male to female courtship assay

DifA-, DifB-, and J4R (control) male flies were collected as pupae, allowed to eclose, and isolated for 4 days at room temperature. The female flies used were virgin Canton S females. The courtship assay was performed in a circular chamber of 0.8-cm diameter. The female was placed inside the chamber and allowed a few minutes to acclimate. The testing male fly was added to the chamber, and courtship was recorded for a period of 10 min. Experimental mutant flies and control flies were interdigitated. The total period of time that the male displayed any of the steps of courtship toward the female was recorded. The courtship index represents the fraction of time the male displayed steps of courtship toward the female within the 10-min period.

### Male to male courtship assay

The male–male courtship assay was performed between a testing genotype and a Canton S male. Both were collected and reared as described for the courtship assay. The Canton S male was marked with a small dot of white paint on the thorax for identification. The courtship assay was performed as above. Wing extension index represents the fraction of time of wing extension by the testing male toward the Canton S male during a 10-min observation period.

### Female receptivity assay

Female receptivity assays used 5-day-old mature virgin females of the experimental and control genotypes, collected as virgins and housed in groups of 10. Males were 5-day-old Canton S males, collected at eclosion, and isolated for 4 days. The female was placed in a courtship chamber, allowed to acclimate for a few minutes, following which the male was added to the chamber. The assay was conducted for a maximum period of 60 min. The assay was terminated if the female mated. The time to copulation was recorded.

### Learning and memory assay

In the learned-suppression-of-phototaxis assay, 5-day-old singly housed males were tested for associative learning in a light–dark choice chamber as described by Le Bourg and Buecher (2002) and modified by Troutwine et al. (2019) with the exception that flies were batch trained (groups of three to five flies) by housing them in a well lit 0.1-M quinine-soaked paper-lined vial for 3 min. Between trials flies are returned to a darkened vial without quinine for 3 min. After three training trials, flies were tested individually in a T-maze for their preference for a lit or darkened chamber using a T-maze. All data is the product of the animals first experience in the T maze. The Preference Index was calculated for a group of three to five flies as [number of of flies that enter the darkened arm] / [number that enter the darkened arm + number that enter the lit arm]. A group of flies that prefers the lit arm will generate a negative preference index while a group with a preference for the darkened arm will generate a positive preference index.

### Circadian rhythm assays

Circadian Rhythm Assay. Flies were raised in a 12:12 light:dark cycle. Single flies were loaded, without anesthesia, into 5 mm x 65 mm glass tubes with food at 1 end (5% sucrose 2% agar) and locomotor activity recorded using the DAM2 Drosophila Activity Monitor System (TriKinetics, Inc., Princeton, MA, USA). Monitors were placed into a 25 °C incubator housed in an unused laboratory. Illumination was 12:12 light:dark for the first 2 days, and then the illumination was extinguished, and the animals were housed in constant darkness for 7 days. The free-running actograms were captured during the constant darkness phase in 1-min bins for the next 7 days. Flies that did not move for any 24-h period were excluded.

The calculation or plotting of actograms, period (chi_sq_periodogram method), and statistical significance of rhythmicity were generated using the Rethomics R package (Geissmann et al., 2019).

### Statistical analysis and graphics

Statistical analysis was performed using R versions 4.3.3 and 4.5.0 (R Core Team, 2021). Plots were generated using the ggplot2 visualization package (Hadley, 2016). Figure 1 was made using the Inkscape program (Inkscape Project, 2025). Statistical tests, number of repeats, and *p* values are included in the figure legends.

## Data Availability

The authors have made the data in this manuscript available at: https://doi.org/10.6084/m9.figshare.30615110.
